# Clinical Surgery of the Hospital of La Pitié

**Published:** 1843-01-21

**Authors:** 


### BIBLIOGRAPHICAL NOTICES.

*Clinique Chirurgicale de l'Hopital de la Pitié.* Par J. Lisfranc. Tome Premier. Paris, 1842. 8vo. pp. 696.

*Clinical Surgery of the Hospital of La Pitié.* By J. Lisfranc. Vol. I. Paris, 1842.

Few medical visitors to the French metropolis will readily forget the distinguished surgeon of La Pitié. The position which he has so long occupied as a clinical professor, the great reputation he has so worthily obtained, and the respect with which his professional opinions are received, render him an object of early interest and attention. His lofty stature, stately gait, and robust form; his large and regular features, his deep set, restless and piercing eye, overshadowed by a heavy brow; his ample forehead, with its few grey locks, surmounted by the old cap; and his pale and haggard expression, all make an appearance eminently striking and not easily forgotten. No man has been more misrepresented than M. Lisfranc. An unfortunate temperament has done much towards injuring his prospects; and to disappointment, acting on a natural infirmity of disposition, may, perhaps, be attributed that eccentricity of character and impetuosity of temper, which are usually associated with his name. His capacity is undoubtedly of an exalted order; surpassed, if equalled, by that of none of his rivals. A quick and vigorous understanding, a lively imagination, a clear and sure judgment, a varied and solid erudition, a persevering industry, and undaunted professional ardour, are his claims to a reputation laboriously and honestly obtained. With these intellectual powers are unhappily combined an aceticism of temper, a violence of prejudice, an impetuosity of feeling, and a selfish spirit, which betray him into the constant indulgence of violent personal invective, to the utter disregard and contempt of the feelings and claims of others. As a lecturer he is full of useful information, which he conveys in a clear, vigorous and energetic style, almost too theatrical to be impressive, and often offensive from self-sufficiency and importance. A consciousness of manifest superiority, and a determined inclination to assert and maintain it, lead him to the constant indulgence of his sarcastic humour, and his sallies indiscriminately directed, are as unmeasured as in general unmerited. Frequently in the midst of a lecture of intense interest he will abruptly stop, his whole frame becoming convulsed, and, without apparent provocation, pour forth, in stentorian voice, a passionate and boisterous philippic on the devoted head of a *confrére*, garnished with admonitory expletives to the neighbouring internes. The fit over, he resumes his discourse, and again interests and delights his class. From his pupils he exacts an entire submission and passive obedience to his whims and caprices, any defection being visited by gross personal abuse, publicly administered. His manner to his patients is usually uncouth and repulsive, he frequently during his morning visits abandoning himself to sudden transports of rage; and in the course of a painful and tedious operation, he commonly exhausts several times his juratory vocabulary. During his life time, Dupuytren was the especial object of Lisfranc's hatred and abuse; since his death he has canonized him and his doctrines, and Velpeau has become the subject of his daily vituperation and invective.

It will be impossible for us to attempt any digest of this volume. An indication of its contents, in a general manner, is the most our limits will allow.

M. Lisfranc devotes a well written preface to the subject of clinical instruction, insisting strongly on its importance. He strenuously combats the old and dangerous doctrine, that it should be regarded as the complement to a medical education, and he thinks that the hospital should early engage the constant attention of the student, in order that he may be able, by practical familiarity with disease, to fully appreciate the theoretical courses and books. As soon as the elements of anatomy are mastered, he recommends an assiduous devotion to clinical pursuits, in order that those subjects which hereafter are to engage attention may be indelibly written on the memory.

> Segnius irritant animos demissa per aurem,
> Quàm quæ sunt oculis subjecta fidelibus, et quæ,
> Ipse sibi tradit spectator.

In systematic books and lectures, all seems simple and uncomplicated, and diagnosis appears invariably easy and certain; whereas, to the most practised surgeon it is often perplexing and obscure. A just valuation of our art is also another consequence of the personal observation of disease. The student is not hereafter obliged to recommence his education; he sees daily the most skilful baffled; he discovers that there are limits to our means, and he does not become disheartened on entering his professional career. M. Lisfranc next passes to the subjects of anatomy, physiology, and pathology, and regards a complete knowlege of these branches as indispensable requisites to the prosecution of clinical research. But these are not sufficient; to ensure the successful application of therapeutics, the nature, progress, symptoms of disease must all be carefully studied. The seat of the disease, temperament, locality, the existence of epidemics, &c., all should be taken into consideration. In medicine, as in everything else, our author thinks, the spirit of system, of exaggeration, and of exclusiveness, has operated injuriously, retarding its progress, and frequently threatening its destruction. A quarter of a century is occupied in erecting and propagating a theory; another, in destroying it. The reason of this is the little attention paid to observation. For most men, theories do not arise from the facts, but the facts are made subservient to the theories. This is the cause of the bitter sarcasms which have been lavished on medicine; and the general want of confidence it has occasioned has largely contributed to the successful career of empiricism. Let us, as far as practicable, adopt in our investigations the rules of the physical and mathematical sciences, and we may yet redeem medicine from the opprobrium by which it is too frequently but justly visited.

The first chapter treats of the uvula. This neglected little organ, the frequent cause of troublesome and obstinate symptoms, often terminating in serious disease, receives at M. Lisfranc's hands full consideration. The next chapter, on the application of leeches, is full of practical hints, which, if more generally followed, would ward off many untoward occurrences. The subject of blood-letting in general is ably handled, and the precepts inculcated of high utility. Fractures, and their treatment, occupy a large part of the volume.

Cancer, the dressing of wounds, the treatment of sprains, burns, venesection at the bend of the arm, the ligature of arteries, necrosis and caries, nervous ophthalmia, tumours of the eyelids, the extirpation of tumours, hernia, ulcers, and white swellings, are each amply discussed, and much that is of a novel and practical nature pleasantly inculcated.

